# Building a FAIR data ecosystem for incorporating single-cell transcriptomics data into agricultural genome to phenome research

**DOI:** 10.3389/fgene.2024.1460351

**Published:** 2024-11-29

**Authors:** Muskan Kapoor, Enrique Sapena Ventura, Amy Walsh, Alexey Sokolov, Nancy George, Sunita Kumari, Nicholas J. Provart, Benjamin Cole, Marc Libault, Timothy Tickle, Wesley C. Warren, James E. Koltes, Irene Papatheodorou, Doreen Ware, Peter W. Harrison, Christine Elsik, Galabina Yordanova, Tony Burdett, Christopher K. Tuggle

**Affiliations:** ^1^ Department of Animal Science, Bioinformatics and Computational Biology Program, Iowa State University, Ames, IA, United States; ^2^ European Molecular Biology Laboratory, European Bioinformatics Institute, Wellcome Genome Campus, Cambridge, Cambridgeshire, United Kingdom; ^3^ Animal Science Research Center, Division of Animal Science and Division of Plant Science and Technology, University of Missouri-Columbia, Columbia, MO, United States; ^4^ Cold Spring Harbor Laboratory, Cold Spring Harbor, NY, United States; ^5^ Department of Cell and Systems Biology/Centre for the Analysis of Genome Evolution and Function, University of Toronto, Toronto, ON, Canada; ^6^ Lawrence Berkeley National Laboratory, DOE-Joint Genome Institute, Berkeley, CA, United States; ^7^ Plant Science and Technology, University of Missouri, Columbia, MO, United States; ^8^ The Broad Institute of MIT and Harvard, Data Sciences Platform, Cambridge, MA, United States; ^9^ Division of Animal Science, University of Missouri-Columbia, Columbia, MO, United States; ^10^ Earlham Institute, Norwich Research Park, Norwich, United Kingdom; ^11^ Medical School, University of East Anglia, Norwich Research Park, Norwich, United Kingdom; ^12^ U.S. Department of Agriculture, Agricultural Research Service, NEA Robert W. Holley Center for Agriculture and Health, Cornell University, Ithaca, NY, United States

**Keywords:** single-cell RNA-seq, metadata, HCA data portal, data ingestion portal, FAANG, data submission standards, data resource and analysis, FAIR

## Abstract

**Introduction:**

The agriculture genomics community has numerous data submission standards available, but the standards for describing and storing single-cell (SC, e.g., scRNA- seq) data are comparatively underdeveloped.

**Methods:**

To bridge this gap, we leveraged recent advancements in human genomics infrastructure, such as the integration of the Human Cell Atlas Data Portal with Terra, a secure, scalable, open-source platform for biomedical researchers to access data, run analysis tools, and collaborate. In parallel, the Single Cell Expression Atlas at EMBL-EBI offers a comprehensive data ingestion portal for high-throughput sequencing datasets, including plants, protists, and animals (including humans). Developing data tools connecting these resources would offer significant advantages to the agricultural genomics community. The FAANG data portal at EMBL-EBI emphasizes delivering rich metadata and highly accurate and reliable annotation of farmed animals but is not computationally linked to either of these resources.

**Results:**

Herein, we describe a pilot-scale project that determines whether the current FAANG metadata standards for livestock can be used to ingest scRNA-seq datasets into Terra in a manner consistent with HCA Data Portal standards. Importantly, rich scRNA-seq metadata can now be brokered through the FAANG data portal using a semi-automated process, thereby avoiding the need for substantial expert curation. We have further extended the functionality of this tool so that validated and ingested SC files within the HCA Data Portal are transferred to Terra for further analysis. In addition, we verified data ingestion into Terra, hosted on Azure, and demonstrated the use of a workflow to analyze the first ingested porcine scRNA-seq dataset. Additionally, we have also developed prototype tools to visualize the output of scRNA-seq analyses on genome browsers to compare gene expression patterns across tissues and cell populations. This JBrowse tool now features distinct tracks, showcasing PBMC scRNA-seq alongside two bulk RNA-seq experiments.

**Discussion:**

We intend to further build upon these existing tools to construct a scientist-friendly data resource and analytical ecosystem based on Findable, Accessible, Interoperable, and Reusable (FAIR) SC principles to facilitate SC-level genomic analysis through data ingestion, storage, retrieval, re-use, visualization, and comparative annotation across agricultural species.

## Introduction

The analysis of how genome information creates phenotypes at the single cell level, the fundamental unit of biology, is a powerful approach for understanding genome function, and is rapidly becoming the gold standard for human genetics research predicting phenotype from genotype. The complex tissues analyzed in traditional genomics research contain many cell types, mixing their gene expression patterns. This makes it hard to see how genes are regulated within specific cell types, and it prevents us from accurately linking genotypes to specific cellular phenotypes. To make the enormous promise of single-cell genomics a reality for the agricultural genome to phenome community, we need to develop Findable, Accessible, Interoperable, and Reusable (FAIR) single-cell data resources, analysis platforms and informatic tools for storing, sharing, and analyzing such data that is currently generated in crop and livestock research groups. Such data is a mixture of all patterns, obscuring regulatory function acting specifically within individual cell types and preventing recognition of the genotype link to cellular phenotypes. Recognition is growing in the agricultural genomics community that genomics data could be more widely re-used if it was more readily available, comprehensible, and easier to integrate with other data (Clark et al., 2020; Harrison et al., 2021). This is especially true for new data types, such as single-cell RNA-seq (scRNA-seq) data providing cellular heterogeneity, which is rapidly expanding across crop and livestock research (Chen et al., 2023; Cole et al., 2021; Herrera-Uribe et al., 2023). The community has numerous data submission platforms for traditional tissue-level analyses available, but little experience in describing and storing single-cell data or meta-data (Wang et al., 2019). The term “metadata” refers to additional details or characteristics linked to individual cells in a dataset (Sheffield et al., 2023). Linked metadata that describes the biological sample from which genomic sequences are derived and analyzed, as well as the processes used to create the data, are crucial for reuse of such data. The establishment of metadata standards plays a pivotal role in the single-cell era and is of the utmost importance for understanding the complexities of SC datasets. Rigorous dataset description enables researchers to derive valuable biological insights such as annotation of individual cell types within tissues through merging disparate data and maximizing power of any inference. However, there are challenges when reusing and integrating SC data. These challenges include; data heterogeneity such as comparison and integration of SC datasets across different studies; inconsistency in experiment protocol and sample handling which may lead to batch effects; annotation discrepancies which hinder accurate comparison of scRNAseq data; lack of comprehensive metadata which is essential for reproducibility; as well as inconsistent sample preparation and sequencing techniques contributes to technical variability which can confound biological signals thus leading to challenges and reproducibility and interpretation of results across different studies (Adil et al., 2021). For example, Grones et al. (2024) focused on following established protocols for data generation, ensuring thorough analysis and quality control, storing data and extensive metadata in publicly accessible databases, and meticulously documenting all procedures and analyses for reproducibility across studies which are necessary to improve transparency and utility in scientific research (Hovig et al., 2021; Harrison et al., 2021; Azevedo and Dumontier, 2020; Jacobsen et al., 2020; Wilkinson et al., 2018; Wilkinson et al., 2019). To improve data reusability, it is crucial to provide extensive metadata including data provenance and clear usage guidelines to evaluate the quality and reliability of data thus aiding in the FAIRification of data (Thompson et al., 2020; Weigel et al., 2020; Wilkinson et al., 2016).

The Human Cell Atlas- Data Portal (HCA-Data Portal)^1^, Single Cell Expression Atlas (SCEA)^2^, Plant Cell Atlas (PCA)^3^, and Functional Annotation of Animal Genomes (FAANG)^4^ efforts are prominent initiatives in the field of single cell genomics, each employing distinct metadata frameworks to facilitate data organization and dissemination (Giuffra and Tuggle, 2019; Jha et al., 2021; Lun et al., 2019; Papatheodorou et al., 2019). There are also new databases that contain data from single-cell based analyses in bacteria for interested readers (Childs et al., 2024). HCA-Data Portal (Regev et al., 2018) is integrated with Terra (Perkel, 2022), a secure, scalable, open-source platform for biomedical researchers to access data, run analysis tools and collaborate. This cloud-based platform is co-developed by the Broad Institute of MIT and Harvard, Microsoft, and Verily, with the goal of accelerating biomedical innovation including scGenomics. Currently, the most comprehensive data ingestion portal for high throughput sequencing datasets from plants, fungi, protists, and animals (including humans) Annotare (Athar et al., 2019), ensures that sufficient metadata are collected to enable re-analysis and dissemination via the SCEA housed at the EMBL-European Bioinformatics Institute (Papatheodorou et al., 2019). Another EMBL-EBI portal limited to animal datasets, the FAANG data portal (Giuffra and Tuggle, 2019; Harrison et al., 2021), provides bulk and scRNA-seq data access which uses a semi-automated process to submit and validate files using the HCA Data Portal (Regev et al., 2018)metadata and data validation service. HCA’s metadata structure encompasses five primary categories: biomaterials, processes, protocols, files, and projects. These categories serve as a comprehensive framework to document and contextualize data. Biomaterials capture information about the biological specimens, while processes detail the experimental techniques involved in data generation. Protocols elucidate the methodologies or procedures employed, and files house the raw and processed data. Projects, as a broad category, provide the research framework and overarching goals. The HCA-Data Portal has been in existence since 2019 and has incorporated 20 million cells from over 2,000 donors, covering 64 organs in the human body, from over 300 labs around the world.

FAANG, in contrast, adopts a FAIR-centric metadata hierarchy that comprises samples, experiments, and analysis. Samples outline the biological origins of an experiment, experiments capture the experimental procedures, and analysis explains the data processing and interpretations. This schema aids in the organizing and monitoring of data as it moves through various data generating and analysis stages. The FAANG data portal itself serves as a centralized, focused resource, offering researchers a wealth of validated, richly described, and high-quality datasets for genome-to-phenome research. Historically, the FAANG data portal has housed tissue-level data, but single-cell datasets are increasing (Herrera-Uribe et al., 2021). The portal offers an API that facilitates comprehensive searches across all FAANG data portal fields, enhancing accessibility and reusability for researchers (Harrison et al., 2018).

The Plant Cell Atlas (PCA), initiated in 2019, functions as another collaborative research coordination network accessible at www.plantcellatlas.org/, that intends to comprehensively describe the state of various plant cell types and aims to integrate high-resolution location information of nucleic acids, proteins, and metabolites within plant cells (Rhee et al., 2019). The recent roadmap paper by the Plant Cell Atlas Jha et al. (2021) delineates the strategic steps towards achieving this ambitious goal. This article and a companion article by Fahlgren et al. (2023) delve into the essential data infrastructure required to support the PCA, addressing critical aspects such as data collection, curation, standardization, integration, and visualization (Ahmed et al., 2021). The articles also explore funding mechanisms for such infrastructure, drawing insights from models utilized by other online resources, though, for now, data standards are being developed as an unfunded community effort. Anticipated data from PCA-associated projects will include initial datasets like single-cell RNA sequencing matrices, subcellular quantitative mass-spectrometry proteomics and metabolomics data, and fluorescent protein (GFP) localization images, and comparative -omics between plant species contributing significantly to advancing our understanding of plant cellular dynamics (Fahlgren et al., 2023).

The Single Cell Expression Atlas, available at https://www.ebi.ac.uk/gxa/sc/home (Papatheodorou et al., 2019) is a valuable bioinformatics resource, providing an integrated online platform comprising a database, user interface, and web service. This resource facilitates convenient access to extensive information on gene expression patterns across diverse species, tissues, cells, experimental conditions, and diseases. SCEA’s metadata framework aligns closely with the AE (ArrayExpress) (Parkinson et al., 2007) platform, adhering to the MIAME (Minimum Information About a Microarray Experiment) (Rayner et al., 2006) and FAIR principles (Wilkinson et al., 2016). Expression Atlas has evolved to incorporate datasets from various reputable sources and repositories, including NCBI’s Gene Expression Omnibus (GEO)^5^ (Clough and Barrett, 2016), the European Nucleotide Archive (ENA)^6^ (Leinonen et al., 2011a), and controlled access datasets like GTEx (Aguet et al., 2020). Currently, the SCEA encompasses data from 12 different species, encompassing not only *Homo sapiens* but also model organisms such as *Mus musculus*, *Arabidopsis thaliana*, and *Drosophila melanogaster.* The curation of metadata is conducted in-house through a semi-automatic process, involving the identification of experimental factors such as cell types, diseases, or perturbations. The existing landscape of single-cell tools for storing metadata includes platforms like Gene Expression Omnibus (GEO) (Clough and Barrett, 2016), ArrayExpress (AE)^7^(Parkinson et al., 2007), Fly Cell Atlas^8^ (Li et al., 2022), HuBMAP^9^, CZ CellxGene^10^ (Abdulla et al., 2023), and scPlantDB^11^ (He et al., 2024), catering to diverse datasets ranging from human and model organisms to livestock and crop data. However, a significant drawback arises from the variability in metadata standards across these databases. The lack of a standardized ingestion method poses a challenge, hindering the integration and visualization of data in alternative portals. Only a few tools are available for agricultural species due to a lack of standardized ingestion methods.

Our overall aim was to determine if the current metadata schema for crop and livestock SC data can be used to ingest an example scRNA-seq dataset in a manner consistent with HCA Data Portal standards and if established resources such as Terra can be used to analyze the ingested animal data. This aim was accomplished through development of specific scripts for processing FAANG data from that portal to the HCA Data Portal. In addition, we created a Terra Workspace which features network analysis of ingested data using GENIE3 algorithm (Huynh-Thu et al., 2010) and a comprehensive interactive application to view network results in a html-based web server file. Terra was specifically chosen for this task because of its robust infrastructure, reusability, scalability, and its integration of the HCA-Data Portal. Although, JupyterLab can be used with Amazon S3 storage (Palankar et al., 2008).

Terra offers several advantages for analysis of single cell data, such as scalable infrastructure, pre-configured workflows, and ready to implement pipelines that reduce setup time and usability across large agricultural community datasets. Our proposed use of Terra’s ecosystem focuses more on the applicability of FAIR data standards to agricultural datasets, while considering the challenges faced within the cloud platforms such as platform lock in and interoperability that often involves upfront duplication of efforts. The Terra infrastructure addresses these concerns by deploying three different cloud-based frameworks [Azure (Wilder, 2012), goggle cloud platform (Bisong, 2019), AnViL (Hall et al., 2023)], significantly reducing the difficulty of integration with other cloud datasets (Sheffield et al., 2022).

In addition, we test and develop prototype tools to visualize the output of scRNA-seq analyses on genome browsers, comparing across tissues and cell populations. Specifically, we introduce a genome annotation browser based on JBrowse that can display single-cell data against gene model annotation in a genome assembly (Buels et al., 2016). This innovative tool takes the output generated from Terra’s analysis pipelines and transforms it into a visually intuitive representation. Researchers can leverage JBrowse to explore single-cell outputs, facilitating the discovery of novel biological insights and promoting data reuse.

## Materials and methods

In this section, we describe methodologies used to ingest and validate the workflow to adapt single-cell livestock data into the HCA-Data portal, primarily focusing on a porcine PBMC dataset. We further tested Terra’s compatibility with the ingested data by carrying out single cell-based network analysis. Additionally, we develop a JBrowse plugin interactive tool to carry out further single cell visualization based on expression level. This section includes datasets, web portals, tools and standards used to integrate to the process of ingestion that can be applied across other agricultural transcriptomics datasets.

### Human cell atlas- data portal workflow validation

#### Single-cell livestock dataset for proof-of principle

The peripheral blood mononuclear cell (PBMC) single-cell dataset was downloaded. The data consists of approximately 28,800 cells from seven separate healthy pigs of various ages analyzed by Herrera-Uribe et al. (2021). This data represents typical data produced within animal genomics community.

#### FAANG metadata schema, rule sets and validation

The FAANG project (Harrison et al., 2021) is a collaborative endeavor spanning multiple global laboratories, committed to generating and interpreting high-quality data across an expanding range of species (Tuggle et al., 2016; Clark et al., 2020). The data for this study was subjected to the well-established FAANG sample and experimental metadata standards and actively promotes best practices in data deposition, description, and openness (Giuffra and Tuggle, 2019; Tixier-Boichard et al., 2021). These evolving standards are continually refined under the guidance of the FAANG Metadata Task Force (Harrison et al., 2021) and version-controlled on GitHub www.github.com/FAANG/faang-metadata with well-documented releases of metadata specifications https://data.faang.org/ruleset/samples#Standard. In this work, we decreased and enlarged the FAANG rulesets to incorporate both scRNA and scATAC datasets, primarily focusing on scRNAseq data shown in Supplementary Table 1. Currently, there is no standardized pipeline allowing automated submission of FAANG data from the FAANG data portal to any resource that allows data analysis beyond simple visualization. The current FAANG metadata standards are organized into three distinct rulesets: sample, experiment and analysis spreadsheets with required, mandatory and optional fields which facilitates streamlined data submission and reuse within the portal. Once the spreadsheets were updated according to new single-cell version, the files then underwent validation and conversion into JSON files which were used to perform ingestion in the HCA Data Portal.

#### HCA metadata schema, data wrangler, validation

The Human Cell Atlas Project is a global initiative with the ambitious goal of characterizing all human cell types based on their unique molecular profiles, including gene expression patterns, and integrating this knowledge with traditional cellular descriptions (Regev et al., 2017). The project defined a highly structured metadata standard with a granular design, on which separate entities represent the stages of the experimental assay (Haniffa et al., 2021). The metadata schema is organized into distinct “types,” stand-alone schemas that define the validation rules for each critical component of experiments. These types include “biomaterial” for biological materials, “protocol” for experimental procedures, “processes” for practical application of protocols, “files” for generated data files, and “project” for high-level, project-specific information. These types have unique fields tailored to describe their respective entities and can include supplementary thematically related fields for flexibility and extensions. Within the framework of the HCA schema modules, “type” schemas encompass a unique set of fields tailored to describe the attributes of the corresponding entity, inheriting fundamental fields from the corresponding “core” entity schema. Additionally, they may include supplementary thematically related fields grouped within Module entity schemas, offering flexibility and extensions related to the specific type (Regev et al., 2017; Regev et al., 2018). Embedded within these thematic modules is a distinct subtype articulated by the HCA schema, namely, ontologies. Through the incorporation of ontology modules, the HCA schema provides the ability to precisely delimit the terms used to define certain attributes (e.g., ethnicity, species) to a set of curated, controlled vocabulary terms, further enhancing the interoperability of the data (Osumi-Sutherland et al., 2021). In the process of metadata submission within the HCA Data Portal, the majority of metadata fields are provided by the data contributors during the submission phase. However, a specific subset of fields within the metadata standard is furnished by the Ingestion Service component of the Data Platform (Rozenblatt-Rosen et al., 2021).

To address the differences between the HCA and FAANG schema, we introduced a pipeline as a proof of principle within the HCA branch that now specifically handles the updated FAANG schema. Subsequently, we adapted and customized available schema and generated entities to integrate the first single-cell porcine dataset into Data Portal ingestion service within the HCA-Data coordination platform framework.

### Terra cost, billing and workspace

We utilized a cloud-based platform to store and analyze ingested pig single-cell data called Terra https://app.terra.bio/ (Perkel, 2022). To tailor secure and scalable environment for data analysis of gene regulatory networks, we configured a Microsoft Azure platform hosted at Iowa State University and a built in workspace titled “AG2PI-ingest”. Terra uses data tables to record metadata, and URI linking to cloud-stored data files, enabling users to easily filter and query massive datasets. This environment is complemented by JupyterLab, which lets users create, execute, and debug code in an interactive notebook configuration with pre-configured tools. Virtual Machines (VMs) provide the computational power needed for analyses, which come in a range of sizes with various sizes available to maximize efficiency and minimize cost. These VMs can be customized with specific software environments.

To test Terra’s compatibility with the ingested data, we implemented an analytical pipeline deploying GENIE3 network analysis tool on Azure platform. We established a virtual environment within Terra workspace through an interactive Jupyter notebook to perform the network analysis. Cloud costs will vary depending on the resources being leveraged, with major sources being cloud storage, compute, and egress (aka. moving data out of the cloud). As an example, the cost of computing and disk usage for our Azure platform-based analysis via an interactive Jupyter notebook on a virtual machine was $4 for 3 days in 2024^12^. GENIE3, developed by (Huynh-Thu et al., 2010), uses regression tree ensembles to predict the levels of gene expression based on the data of other genes. The GENIE3 tool identified interactions between transcription factors (TF) and target genes (TG) particularly in the CD4^+^ T cell type, with measured importance scores thereby treating each gene as a target variable to identify which genes best predict its expression. We used python programming language to predict the networks and downloaded the transcription factors from animalTFDB (Hu et al., 2019) database and the target genes from the ingested dataset. Consequently, the results were plotted using the Pyvis library in Python, which was used to develop the web interface for displaying the results generated by GENIE3 (Perrone et al., 2020). Terra’s workspace configuration not only allows for detailed analysis but also supports reproducibility as all users/collaborators have access to the shared workspace.

In addition to storing data, Terra’s public cloud infrastructure facilitates easier organization, access and analysis by allowing users to share workspaces with collaborators. Workspaces combine data, metadata, and analysis tools into a secure cloud workbench. The platform provides three distinct access levels for a workspace, namely, a writer, owner, and reader, each having a unique set of permissions. WDL scripts enable workflows in Terra that can be written by users or transferred from resources such as Dockstore (O’Connor et al., 2017) hence improving the reproducibility and accessibility of research^13^.

### EBI single-cell expression atlas workflow validation

#### Single-cell dataset for plant and animal data ingestion

Plant single-cell expression data derived from 20 experimental studies from 4 major plant genomes (*A. thaliana*, Oryza sativa, Solanum lycopersicum, Zea mays) (Supplementary Table 2) has been ingested into the EMBL-EBI single-cell expression atlas (Papatheodorou et al., 2019) in close collaboration with the Gramene^14^ (Tello-Ruiz et al., 2021). After the extensive manual metadata curation and quality checks by experts, this data is displayed for users on the EMBL-EBI single-cell expression atlas site (www.ebi.ac.uk/gxa/sc) to explore through dimensionality reduction plots, gene expression heatmaps and marker genes. This data will also be indexed and accessible at cellular level via an embedded Atlas widget within Gramene’s search browser https://www.gramene.org/in the future. Similarly, animal single cell expression data was derived from existing study of PBMC porcine single-cell dataset (Herrera-Uribe et al., 2021). The data consists of approximately 28,800 cells from seven separate healthy pigs of various ages and has been ingested to EMBL-EBI single-cell expression atlas.

#### Single cell expression atlas data ingestion

To foster integration with various model organism communities and ensure the harmonious comparison of datasets generated across multiple laboratories, a concerted effort has been made by SCEA to standardize metadata and the requirements for raw and processed data (Wilkinson et al., 2016). Moreover, for technological metadata encompassing facets like library construction, cell isolation, and cDNA amplification, new Minimum information for Single Cell experiments (MinSCe) and previous MAGE-TAB standards have been established. These standardized terms have been thoughtfully integrated into the Experimental Factor Ontology (EFO) (Malone et al., 2010), with unique labels assigned to each entity, further promoting consistency and clarity in metadata representation. We closely worked with the Gramene collaboration and EMBL-EBI for the ingestion of bulk and single-cell plant and animal data, to ingest various datasets in the portal. Furthermore, we used their existing ingestion pipeline to add the first pig single-cell datasets to the SCEA portal. A reproducible scRNA-Seq dataset comprises three fundamental components: raw data, processed data, and metadata, which serves to describe and link to the raw data. This cohesive framework is followed by the data submission tool, Annotare (Athar et al., 2019) or the existing datasets which are extracted from GEO/AE. Upon the data submission process’s completion, a rigorous manual review is undertaken to ascertain the completeness and integrity of the raw data and metadata. In the latest release of the SCEA (Moreno et al., 2022), a comprehensive suite of visualization tools is offered to empower users to explore cell clusters, gene expression levels, and the values of metadata fields. This view reveals either the author’s inferred cell types, if available, or, in their absence, the atlas-calculated cluster annotations at an intermediate resolution value. An important addition to this release is the computation of marker genes, now available not only for the author’s inferred cell types but also for the cell clusters determined by SCEA. This enhancement further enriches the analytical capabilities of the platform, enabling users to gain deeper insights into cell heterogeneity, gene expression patterns, and the biological underpinnings of the datasets.

### Additional tool development for interactive visualization: JBrowse genome browser

To facilitate the visualization of scRNA-seq-based expression levels in a genomic context, we developed a plugin for the JBrowse genome browser (Buels et al., 2016). The test dataset used for development was the same PBMC dataset used in the development of the data ingestion workflow (Herrera-Uribe et al., 2021). We developed the test genome browser using the Sscrofa11.1 genome assembly and the associated gene annotation from Ensembl Release 97. Starting with a Seurat object (RDS format), we used the ExportToCellbrowser function in Seurat 3.1.4 to export an expression matrix (exprMatrix.tsv) and a cell metadata file (meta.tsv), which are formatted for use with the UCSC Cell Browser (Speir et al., 2021). We then used utilities available from the UCSC Genome Browser website (Raney et al., 2024) to further format the data to create an input file for a genome browser track. First, matrixClusterColumns was used to combine the exprMatrix.tsv and meta. tsv files, creating a new expression matrix containing cell clusters or types as column headers. Then matrixToBarChartBED was used with the new expression matrix and BED file of gene annotations to convert the expression matrix into BED6+4 format containing gene identifiers, gene locations, gene names and their expression levels for each cell type. The resulting BED file was sorted using bedSort, and a bigBED file was created using bedToBigBED. Code for the JBrowse track plugin was developed using guidelines provided on the JBrowse website https://jbrowse.org/docs/plugins.html and by investigating existing JBrowse plugins. We developed and tested the plugin using JBrowse Release 1.16.11, as implemented with Apollo 2.7.0 (Dunn et al., 2019).

In conclusion, distinct portals that were used above to develop an ingestion framework enhances the findability, accessibility, interpretability and reusability of single cell transcriptomics datasets across different livestock species along with interactive visualization. This will also facilitate the future ingestion and computational workflow developments within the plant single cell community.

## Results and discussion

In this section, our purpose was to construct an infrastructure to leverage the well-established single-cell metadata standards within the agricultural genomics community for enhancing data reusability along with testing the compatibility of the associated computational environment. We aimed to develop pipelines for ingestion and validation of livestock data into globally recognized tools such as the services and infrastructure supporting the HCA-Data Portal and SCEA. By doing so, this reuse will enhance the single-cell infrastructure for agricultural species, fostering improved data interoperability and accessibility across diverse genomics platforms.

### Improvement in animal workflow pipeline: FAANG data portal to HCA data portal workflow development

**FIGURE 1:**
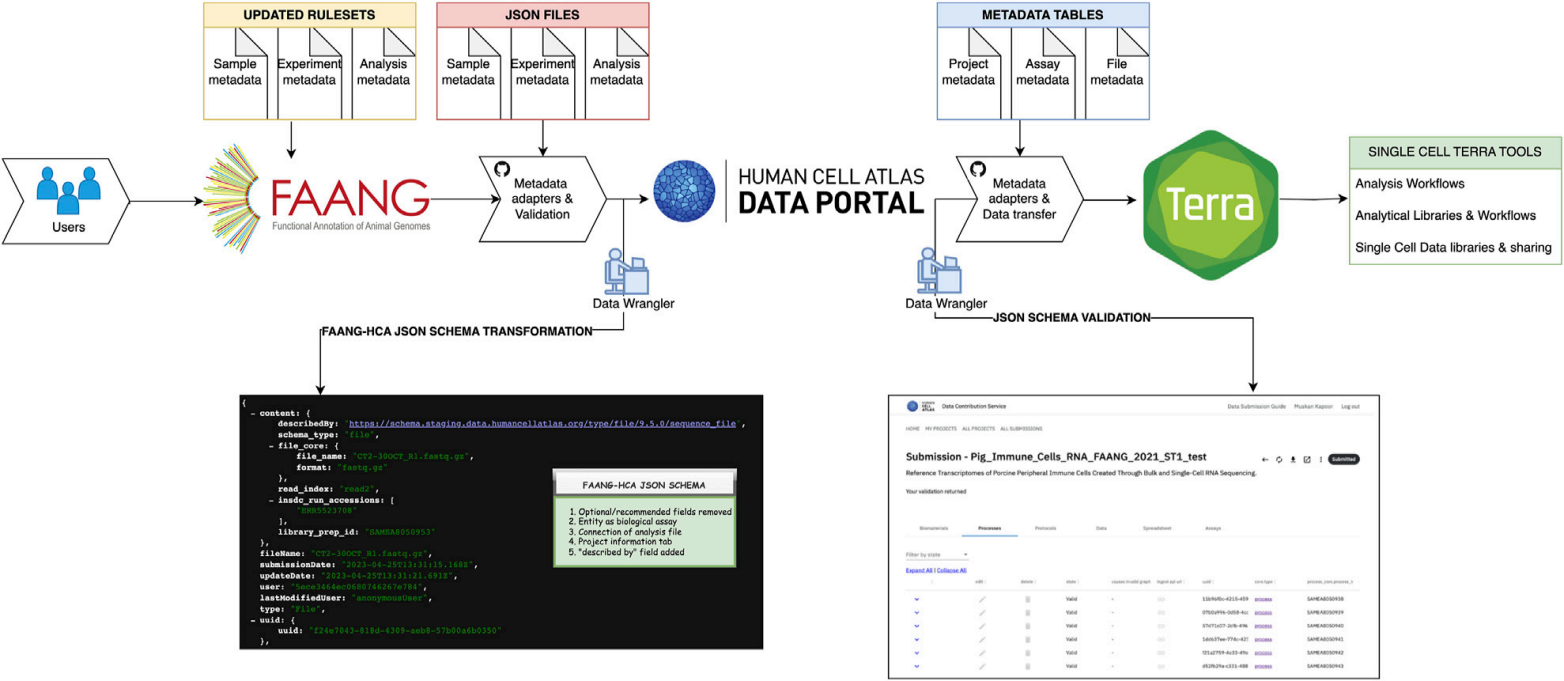
Ingestion in HCA Data-Portal; First result of FAANG-HCA JSON schema is one of the entities in the API, showing the transformation in the format by data wrangler employing metadata adaptors and validation checks. The points (1–5) in FAANG-HCA JSON schema shows the steps optimized to make data compatible and validated in the HCA ingestion service. The second result is JSON validated schema by metadata adaptors in HCA-Data Portal which shows an image of the UI of validated entities along with data transfer into Terra by data wranglers.

We opted to utilize a porcine scRNA-seq dataset (Herrera-Uribe et al., 2021) as a proof of principle of the workflow developed to move data from FAANG data portal to the HCA Data Portal. We aimed to develop pipelines for ingestion and validation of livestock data into globally recognized tools such as the services and infrastructure supporting the HCA-Data Portal and SCEA. By doing so, this reuse will enhance the single-cell infrastructure for agricultural species, fostering improved data interoperability and accessibility across diverse genomics platforms. Thus, we opted to utilize a porcine scRNA-seq dataset (Herrera-Uribe et al., 2021) as a proof of principle of the workflow developed to move data from FAANG data portal to the HCA Data Portal. This dataset serves as a representative example of the data generated by the animal genomics community, illustrating the practical application of our prototype in real-world scenarios. Currently FAANG portal only has rulesets for bulk RNA seq, therefore we undertook the task of updating the rule sets for single-cell data using the existing metadata structure in the FAANG portal, encompassing both scRNA and scATAC data. To align these datasets with the current metadata standards, we established new descriptors for samples, experiments, and analysis spreadsheets in accordance with the FAANG metadata schema as shown in Supplementary Table 1. Subsequently, we created JSON files from the updated spreadsheets and subjected them to validation using the FAANG validation system. In our proof-of-principle for data ingestion, we ingested the FAANG-validated files, adapting non-human schemas via the HCA Data Portal ingest platform as illustrated in Figure 1. The JSON schema for the ingestion of FAANG data into the HCA Data Portal can be accessed through this link: https://github.com/ebi-ait/ag2pi-2-ingest/tree/main/json_schema During the ingestion process, we encountered certain discrepancies arising from differences in schema, metadata adaptors and validation services between the two portals.

To address these challenges, we took a systematic approach which would require a data wrangler (Figure 1). We established a separate branch for the proof of principle ingestion, named the FAANG to HCA branch, and made the source code available on this GitHub Link: https://github.com/ebi-ait/ag2pi-2-ingest. This allowed us to streamline our efforts to harmonize the data and metadata. Our first challenge for FAANG to HCA JSON schema transformation centered around the categorization of fields in the FAANG schema, which includes “optional,” “recommended,” and “mandatory” values. In contrast, the HCA JSON schema primarily covers “mandatory” and “optional” fields, not allowing non-defined attributes to be passed down. To resolve this, we excluded the “recommended” category and updated the FAANG rule sets and JSON files accordingly to align with the new schema. Therefore, for all new incoming datasets we changed the rulesets of FAANG-HCA JSON schema to include only “mandatory” and “optional” fields to be further ingested within the HCA-Data Portal.

The second challenge arose when translating the documents (metadata files) generated by FAANG into documents that could be ingested into the HCA Data Portal ingest platform. As previously mentioned, the HCA portal data model is composed of experimental entities, linked together to form graph structures that represent biological assays. On the other hand, FAANG organizes the data in a slightly more compact data model, where only three JSON files are generated: analysis, samples, and experiment information. To tackle this challenge, we designed a script to translate these three types of files into separate entities that could be ingested and would address the generated FAANG-to-HCA schemas https://github.com/ebi-ait/ag2pi-2-ingest/blob/main/src/utilities/clean_data.py. For any new FAANG ingested dataset, the analysis, samples and experiment information metadata files will be divided into separate entities to match HCA-Data Portal ingestion schema.

Subsequently, as the FAANG data portal allows analysis sequence files submission to ENA, there was no connection to samples and experiment files as required by the HCA schema. Thus, our third challenge centered around establishing such connections between the analysis files and other related components. This information is collected in the metadata in FAANG via connection attributes, whereas the HCA portal schema links the assay parts via database linking of entities. We wrote several adaptation scripts to translate these relationships into database links, connecting the organism, specimen and file metadata in the database https://github.com/ebi-ait/ag2pi-2-ingest. This is shown as a JSON format image in Figure 1, which is an analysis FAANG adapted JSON file translated into new FAANG to HCA schema. Henceforth, FAANG validated datasets will have a connection between all three files to be directly ingested within HCA-Data portal to facilitate automated curation.

A fourth challenge emerged from the absence of a project information tab in the FAANG background files. The HCA Data Portal schema relies on this tab to contain critical information for data search and extraction. To resolve this, we manually collected and introduced the project information in our ingestion script from the existing accession sources, such as ENA, the FAANG portal and the (Herrera-Uribe et al., 2021) metadata of seven PBMC dataset used, effectively generating a comprehensive project information tab aligned with HCA data ingestion project and the metadata standards.

Lastly, the fifth challenge involved a critical component in the HCA-Data portal schema, the self-describing attribute “described_by”. This field points to the JSON schema URI and serves as a way for each of the documents to include information about what fields can/must be included, alongside description and validation rules. To address this, we laid out the schemas in a public GitHub repository and included the field referencing the JSON documents in this repository https://github.com/ebi-ait/ag2pi-2-ingest/tree/main/examples. While solving these challenges, we also designed the first steps into making a sustainable, interoperable process, by identifying and designing solutions that could be applied to future ingested datasets, should they need to be ingested by other data wranglers until the full infrastructure can be adapted.

The metadata schema, aligned with HCA Data Portal standards, aided the data ingestion as well as validation into the HCA Data Portal ingestion service through the HCA-JSON schema validation illustrated in Figure 1. To achieve a reproducible metadata transfer for every dataset, we established a dedicated GitHub page under AG2PI - > Ingest, accessible under the EMBL-EBI-AIT organization at https://github.com/ebi-ait/ag2pi-2-ingest, utilizing Python scripting as an initial proof of concept. Initially, a script was developed to refine the metadata, enabling operations such as cleaning, deletion, or addition of files and attributes in JSON format according to the FAANG-to-HCA Data Portal JSON schema. Furthermore, comprehensive guidelines were provided on the GitHub page, offering a step-by-step description for creating a new submission adhering to FAANG-to-HCA Data Portal standards. This ensures a standardized and reproducible process for further testing while the infrastructure is adapted to accommodate for future datasets. The GitHub page also features the creation of a valid graph within the HCA Data Portal, illustrating the interlinkages among files and the flow of the data ingestion process. The HCA Data Portal ingestion platform provides supplementary metadata, required for systems to understand, classify and validate the data, such as schema version (to understand which set of rules entities were validated) and UUID (Unique Universal identifier), amongst others. To enhance the FAANG-validated JSON files, we introduced these additional fields that aimed at establishing connections between the files and ensuring their successful validation, reinforcing the comprehensive nature of the metadata ingestion process. Furthermore, a script was implemented to generate a new spreadsheet, facilitating user downloads or downstream analysis. This step enhances the accessibility of the data for users who wish to delve deeper into its specifics. Following the successful ingestion and showcasing of the data on the HCA Data Portal ingestion service, the subsequent phase involves transferring the data to a computing environment. This transfer is executed using a curl command, providing access to a plethora of single-cell tools. Users can either employ existing pipelines or create customized ones for further analysis, unlocking the potential for discovering new biological insights.

### Terra workflow for gene regulatory network analysis using GENIE3

**FIGURE 2:**
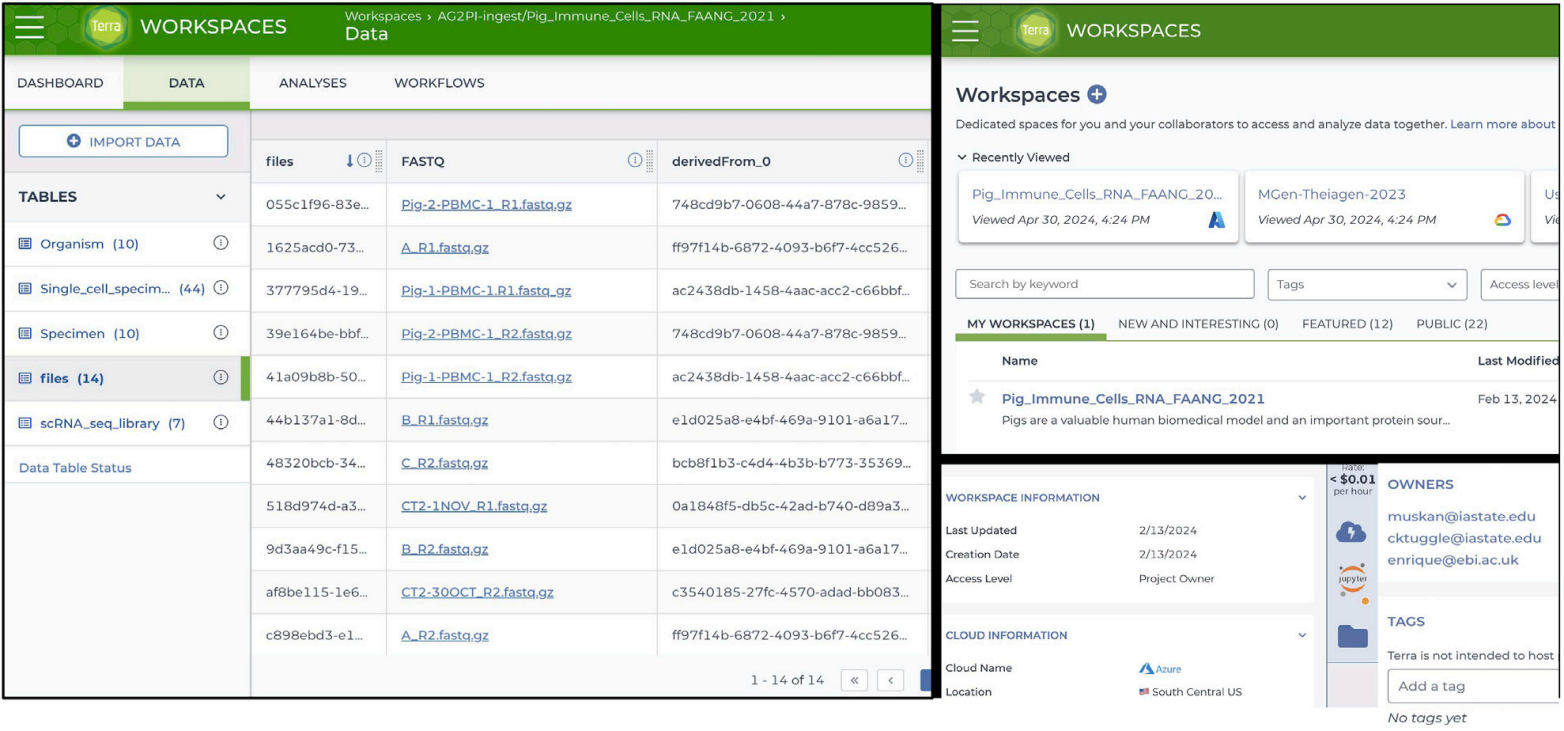
Terra Workspace Dashboard for AG2PI-Ingest showing metadata tables linked to cloud native file storage (left), workspace faceted search (upper right), and cloud computing environment detail displays for cloud transparency (bottom right).

**FIGURE 3:**
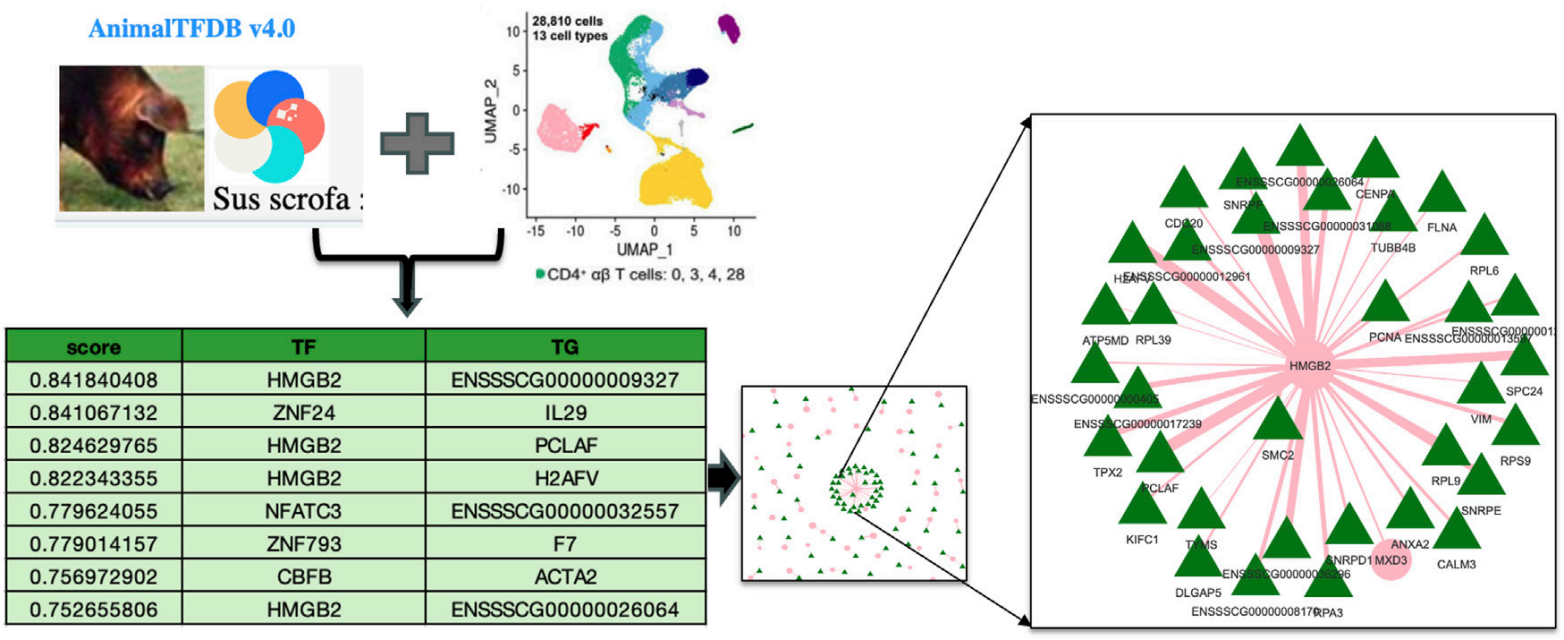
Analysis using Terra workspace for gene regulatory network analysis deploying GENIE3 algorithm for the ingested porcine scRNA-seq data. The figure highlights the interactions between transcription factors (TF) and target genes (TG) particularly in CD4^+^ T cell type, with measured importance scores showing the ranked interactions of each TF on its target genes.

To utilize the Terra environment once the porcine PBMC data was ingested and validated, a comprehensive gene regulatory network analysis was conducted. A new workspace named “AG2PI-ingest” was then created, and writer, reader or owner access was granted to the collaborators along with the metadata tables, which can be imported and reviewed in the workspace under data tables column as illustrated in the left of Figure 2. For the analysis of the ingested data, a virtual environment was established within an interactive Jupyter notebook which employed GENIE3 algorithm showing significant TF associated with the TG and an importance score, influencing gene expression in the CD4+_T cell type of the healthy PBMC data. Importance scores/weights are computed by averaging across all trees in the model and are quantified by metrics such as Gini importance. This constructs key regulatory networks with rankings and an importance score for every TF and their TG. As demonstrated in Figure 3, prediction from table shows the scores of top 8 target genes along with their transcription factors with measured importance scores as representation of their ranked interactions.

We chose the specific TF network for HMGB2 because it contained the strongest hub gene. Moreover, the involvement of HMGB2 in T cell transcriptional regulation is known (Neubert et al., 2023). This is now illustrated for pig cells for the first time using an interactive web-based HTML file represented in Figure 3. This analytical workflow involves using a Jupyter notebook within Terra to analyze the ingested data, visualize the network and create an example workspace, which demonstrates how to manage cloud resources and computing environment along with infrastructure and other costs involved which in our case amounted to approximately $4 for this specific analysis as indicated above. The link and documentation to the Jupyter notebook analysis is at GitHub repository for further reuse https://github.com/kapoormuskan/Terra-Analysis. Additionally, the computational environment within the Terra platform provides functionalities such as pausing, ending, or initiating new environments, which helps manage associated costs effectively.

### General route of metadata flow for current and improved workflows in plant, animal and public databases

**FIGURE 4:**
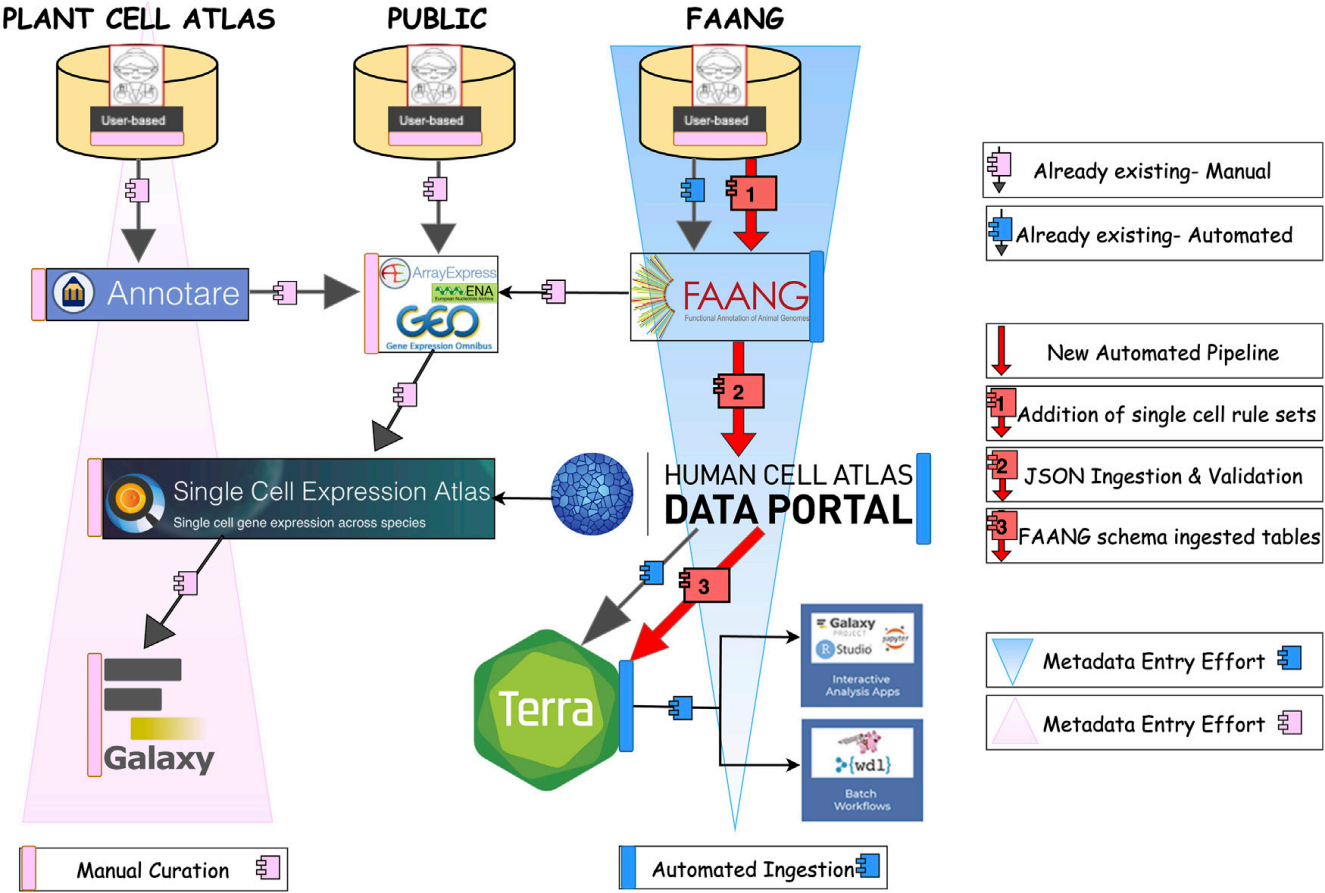
The general route of meta-data flow in Plant cell atlas, public, and Animal. The black arrows with pink and blue boxes shows existing manual and automated path to the exsisting pipelines. Whereas the red arrow with numbering shows the steps we employed to achieve an automated pipeline from FAANG portal to HCA- Data portal from which ingested data is then transferred and shown in Terra.

In single-cell RNA sequencing (scRNA-seq), the general workflow now unfolds through three different paths, namely, datasets submitted on plants through a community such as Plant Cell Atlas, datasets submitted on farm animals through the FAANG community which is focusing on data standardization across diverse animal species, and the domain of public data like AE or GEO (Figure 4). Different communities of researchers approach data archiving, processing, and metadata reporting using various strategies.

**FIGURE 5:**
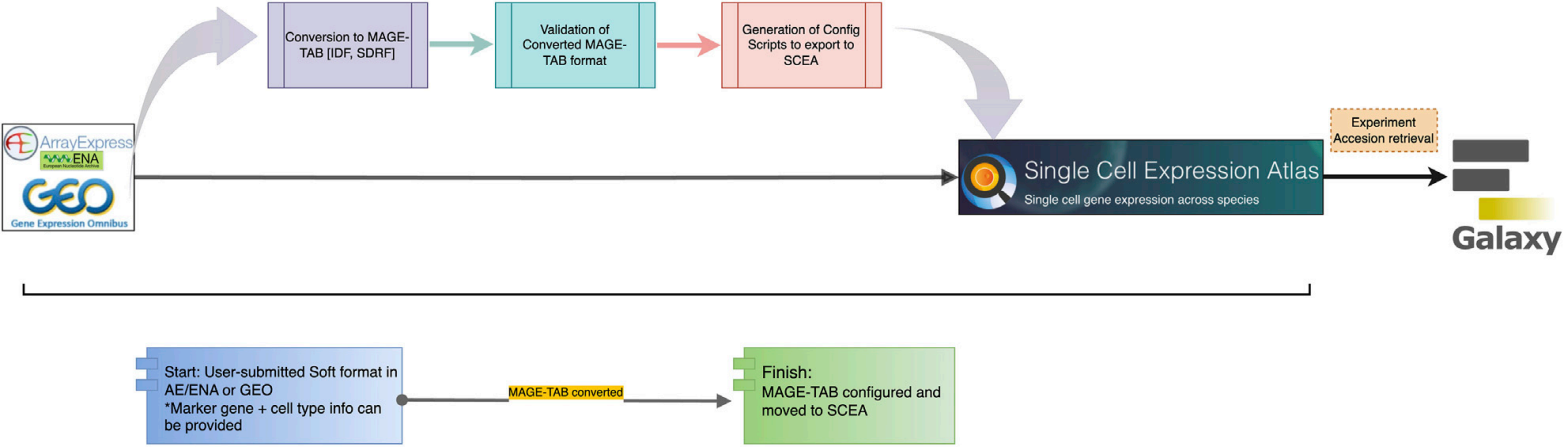
Ingestion of Plant Side, the Single-cell data from public archives follows a route through three scripts. The data can be visualized in the SCEA portal itself and analyzed in the GALAXY through web API retrieval.

In the plant community, where these tasks are not tightly coordinated, this usually entails researchers depositing raw data in large public nucleotide repositories [NCBI SRA (Leinonen et al., 2011b), EBI ENA (Leinonen et al., 2011a)], processing their data using *ad hoc* scripts that utilize various available R or python packages, and hosting processed data and experimental and inferred metadata either as Supplementary Tables accompanying the manuscript describing the experiments, on public expression repositories such as the Gene Expression Omnibus (Clough and Barrett, 2016), FigShare (Thelwall and Kousha, 2016), and SCEA (Papatheodorou et al., 2019) or on researcher-curated webpages (e.g., scPlantDB) (He et al., 2024). Within the existing SCEA framework, the path involves time-consuming manual curation, and the metadata submission which takes place in Array Express (Parkinson et al., 2007) with minimal annotation or in SCEA specific data submission tool called Annotare (Athar et al., 2019). The pipeline initiates by sourcing the raw sequencing dataset from public archives, such as NCBI’s Gene Expression Omnibus (GEO) (Clough and Barrett, 2016), BioStudies (Sarkans et al., 2018), and the European Nucleotide Archive (ENA). A critical step of data curation and validation is carried out by the curators employing a suite of in-house scripts. In Figure 5, the data acquisition from GEO is depicted, followed by the execution of semi-automated scripts by the curators. These scripts play a crucial role in converting the existing standards into a MAGE-TAB format, comprising IDF and SDRF files. Upon the successful conversion of files, subject matter experts (SMEs) conduct thorough file checks and map ontology terms to describe the entities and allow comparison of the same cell type across multiple datasets. Additional information is incorporated into both IDF and SDRF files specifically tailored for single-cell data. Subsequently, SMEs perform validation checks on the converted data. Once the validation process is successfully completed, configuration scripts are generated. These scripts serve as the basis for transferring the data to SCEA and are executed by the SMEs at EBI. This meticulous process ensures the integrity and quality of the data, adhering to standardized formats and facilitating its integration into SCEA portal for further visualization processes and consequently downstream analysis in the Galaxy (Tekman et al., 2020) computing environment. To facilitate user access and analysis, tools like Galaxy are available within the SCEA portal, enabling users to conveniently retrieve metadata via accession numbers and conduct data analysis and visualization. This integrative approach underscores the significance of robust data management and accessibility within the scRNA-seq landscape, ultimately advancing our understanding of single-cell biology.

For livestock, most of the available data has followed the same path as for crops above; submission to ENA or NCBI with minimal metadata descriptions. However, for FAANG, the path of scRNA-seq data and metadata is now submitted through a predominantly automated process, which is preferred due to increased throughput as well as efficiency and a main objective of our study. This pathway now involves the addition and submission of new single-cell rulesets and raw sequencing data to the FAANG and ENA portals. Data flows from the FAANG portal to the HCA Data Portal ingestion process through automated data ingestion, and further validation is performed during the flow to Data Portal ingestion service. Furthermore, the dataset is automated to be effortlessly incorporated into EMBL-EBI’s ingestion service and subsequently transferred to Terra, facilitated by an automated curl command by the data wranglers. Within the Terra environment, researchers/users can find dedicated workspaces and repositories optimized for single-cell analysis. These resources house a suite of powerful single-cell analysis and visualization tools, fostering collaboration and knowledge sharing among diverse scientific communities.

In the context of public data within the scRNA-seq domain, the workflow closely parallels that of the plant pathway (Papatheodorou et al., 2019), with the primary distinction in the origin of the data, which predominantly derives from pre-submitted datasets such as ENA/GEO or AE (Clough and Barrett, 2016; Leinonen et al., 2011a; Parkinson et al., 2007). These datasets typically adhere to the MAGE-TAB format (Rayner et al., 2006), a standard in the field. Rigorous conversion and validation checks are conducted to ensure that the formatted data aligns with the SCEA portal. The validation process is executed by the curators in the EMBL-EBI using specialized scripts. Once the validation is completed, data is transferred to the SCEA which provides a conducive environment for improved visualization, enhanced data reuse, and secure long-term storage.

### JBrowse genome browser track

**FIGURE 6:**
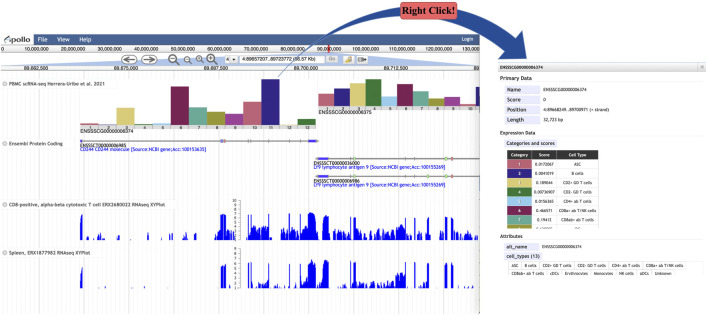
**(A)** JBrowse view showing tracks for PBMC scRNA-seq, Ensembl protein-coding genes, and two bulk RNA-seq experiments. The scRNA-seq track shows a bar chart for each gene, depicting gene expression levels for each cell type. **(B)** A detailed panel, available by right clicking a bar chart, provides a table called “Categories and Scores” showing the gene expression level (called “Score”) for each cell type.

We wished to enhance the capability of JBrowse (Buels et al., 2016), a widely used genome browser platform, to display scRNA-seq-based expression data in a genomic context, similar to the scRNAseq tracks available in the UCSC human genome browser (Raney et al., 2024), so we developed a JBrowse plugin called “BarChartViewer”. By implementing this plugin in a JBrowse1 instance, tracks can be created using scRNA-seq-based expression data in BarChartBED or BarChartBigBED format. The track provides a histogram for each gene showing expression levels for each cell type (Figure 6A) in a tissue sample. The width of each histogram spans the length of a gene, and it adjusts in width according to the genome browser zoom level. Right clicking a histogram opens a window showing details including a table with expression values and cell types (Figure 6B). scRNA-seq expression data tracks provide an additional layer of annotation that can be viewed in the genome browser in the context of many other genome annotation data types, such as bulk RNA-seq and functional annotation data. If multiple samples of the same tissue are available, sample-specific scRNA-seq histogram tracks allow users to quickly compare relative cell-type expression levels for the same gene. The plugin code and the current version 1.0 is accessible by clicking on “Releases” on GitHub https://github.com/elsiklab/BarChartViewer with the example track data and track configurations.

To conclude, the outcomes from our data ingestion pipeline and subsequent analysis within the computational environment of Terra represent significant advancements particularly in the FAIR standards within the agricultural genomics community. The demonstrated successful adaption of HCA metadata schema aligning with livestock schema provides a refined data ingestion framework, which will significantly contribute to improving the accessibility and utility of single-cell data in future genomics research. This will further ensure collaborative and accessible computational framework within Terra providing access to worldwide researchers aimed at improving the understanding/cellular heterogeneity of single-cell data.

## Conclusion and future directions

**FIGURE 7:**
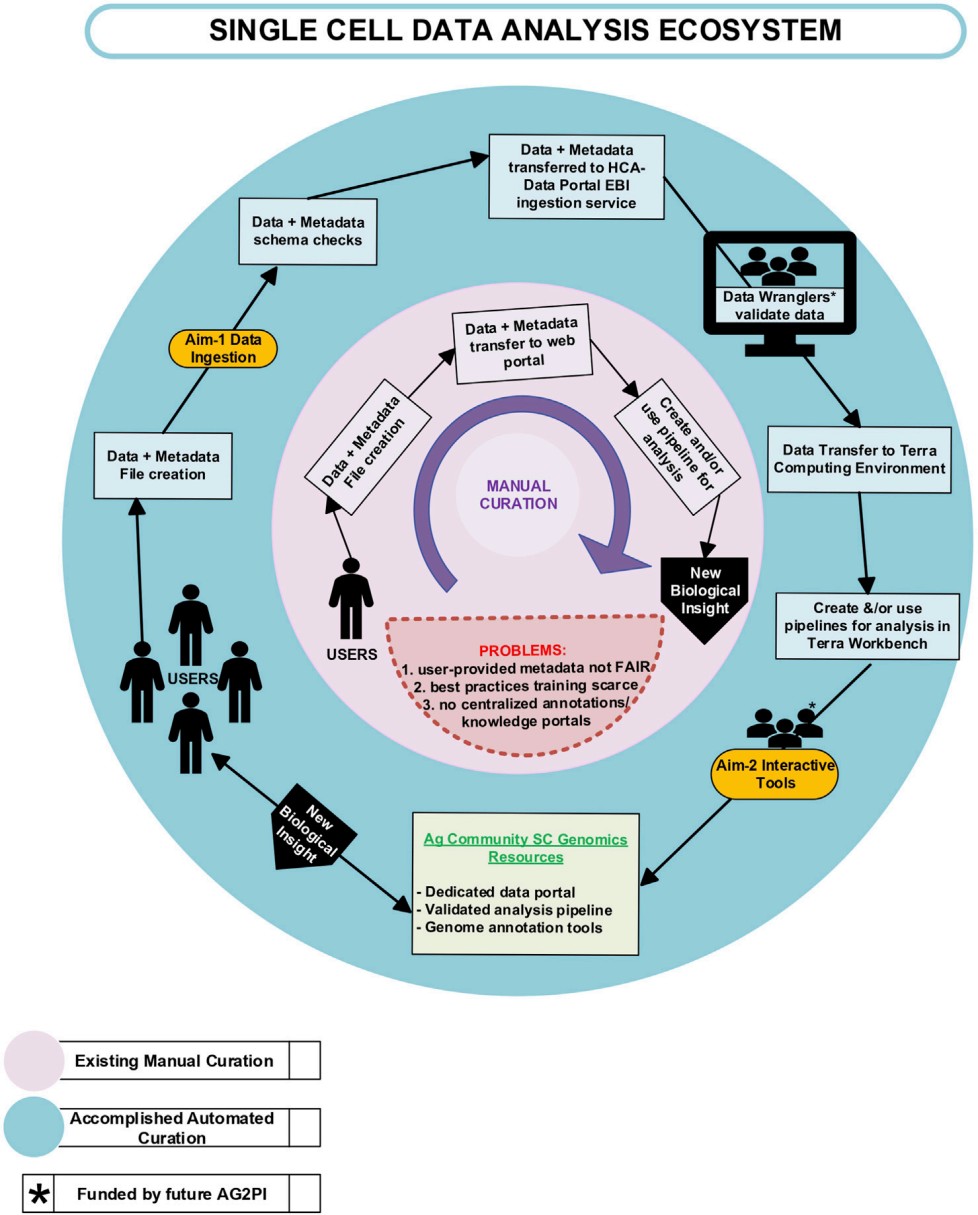
Current Status describing the single cell data ecosystem when the data and metadata file is created and transferred to a web-portal with or without the help of curators. Inner circle (in pink) represents the existing problems with manual curation and the outer circle (in blue) represents FAIR data with automated curation along with interactive scGenomics tools.

This study aimed to address the challenges associated with metadata standards in agricultural genomics by exploring the potential value of integrating single-cell data flow from the FAANG data portal into well-established infrastructures supporting single-cell research such as the HCA Data Portal and SCEA. We present a comprehensive overview of our approach, emphasizing the importance of structured metadata in ensuring Findability, Accessibility, Interoperability, and Reusability (FAIR) principles in data management. The present workflow in Figure 7 (outside ring, currently in place only for FAANG data types) illustrates our proposed approach to data ingestion, validation, and analysis, showcasing the potential for integration of agricultural genomics data into established single-cell research supporting infrastructures. The inner circle (in pink) provides a schematic depiction of the process available prior to this work, which involves data transfer to a web-portal, which are often maintained by individual laboratories, where it can be used for visualization or analysis. However, this *ad hoc* process can create problems with data or metadata not being FAIR and lacking centralized annotation or knowledge portals. In contrast, the details in the outer circle (in blue) shows the specifications that define our vision to address these deficits, including submission of data and metadata to various single-cell resources, such as the HCA Data Platform and SCEA. Users are helped by dedicated data wranglers and curators who ensure the complete and accurate automated flow of information. Upon submission, the data undergoes a rigorous validation process conducted by the data wranglers. This critical step ensures that the ingested data and metadata are well-structured and validated, meeting the community standards of metadata quality. Subsequently, the validated data is submitted either by a user or automatically from a portal (e.g., FAANG) to the HCA Data Portal ingestion service. Once within Terra’s computing environment, users gain access to many pipelines designed to simplify and enhance data analysis. Terra offers a diverse array of single-cell analytical pipelines, complete with example workflows, making it a valuable resource for researchers in the field. Our overarching goal was to determine the adaptability of the current metadata schema for crop and livestock data to scRNA-seq datasets, aligning them with HCA portal standards and further testing Terra compatibility. Looking forward, our work could lead to advancements towards metadata standardization across agricultural platforms which will enable better collaboration between data curators and other scientific communities to refine such future metadata schemas. Thus, our improved ecosystem benefits the users by providing access to high quality metadata for human and other species, automated curation of datasets, simplified data submission and improved findability of distinct agricultural scRNAseq data. Agricultural researchers can utilize the HCA-Data portal framework to gain access to advanced sc tools, workflows and computational nodes that can also directly use human/mouse data to perform comparative analysis, integration, and other downstream applications. Such capability will shift the focus of the single cell community in agriculture from deposition or handling of datasets more into analysis and interpretation. In addition, the development of prototype tools, including a new single-cell track plugin for the genome annotation browser JBrowse, enhances data visualization and exploration. This JBrowse plugin allows researchers to explore cell type expression levels per gene in a genomic context. Furthermore, this JBrowse instance can be utilized to improve context visualization to enhance interactive data analysis.

However, additional future work by the community is required for an optimal environment which will include improved portability of sc metadata files into computational frameworks to ensure data and built-in workflows can be efficiently transferred and analyzed across different cloud platforms. Accessibility across cloud platforms is required to develop user friendly interface, validated workflows and analytical tools. These utilities can further be expanded beyond RNA to other multi-omics studies.

Envisioning the future of agricultural single-cell genomics, we expect to see a shift towards cloud-based analysis platforms which for biomedical research currently enables a scalable and secure environment. Cloud computing infrastructure is mainly dominated by Google Cloud Platform (GCP), Microsoft Azure and Amazon Web Services (AWS) (Narula et al., 2015; Geewax, 2018; Wilder, 2012). Acknowledging the potential of cloud computing, Broad Institute of MIT and Harvard, Microsoft and Verily co-developed and launched the Terra native platform (Perkel, 2022). Terra is operational on both GCP and Azure (Duyzend et al., 2024) which focus on data access and sharing, scalable analytical tools, and robust security measures. Finally, our study contributes to advancing the field of agricultural genomics by integrating robust data management, validation, and analysis within a collaborative and accessible framework.

## Data Availability

Publicly available datasets were analyzed in this study. This data can be found here: https://www.ebi.ac.uk/ena/browser/view/PRJEB43826 European Nucleotide Archive, PRJEB43826.

## Footnotes

1
https://www.humancellatlas.org/

2
http://www.ebi.ac.uk/gxa/sc

3
https://www.plantcellatlas.org/

4
https://www.animalgenome.org/community/FAANG/

5
https://www.ncbi.nlm.nih.gov/geo/

6
https://www.ebi.ac.uk/ena/browser/

7
https://www.ebi.ac.uk/biostudies/arrayexpress

8
https://flycellatlas.org/#tissues

9
https://portal.hubmapconsortium.org

10
https://cellxgene.cziscience.com/gene-expression

11
https://biobigdata.nju.edu.cn/scplantdb/home

12
https://support.terra.bio/hc/en-us/sections/10090806360475

13
https://support.terra.bio/hc/en-us/articles/13184974155803-Cloud-environment-Persistent-Disk-storage

14
https://www.gramene.org/

## References

[B1] Abdulla S. Aevermann B. Assis P. Badajoz S. Bell S. M. Bezzi E. (2023). CZ CELL×GENE Discover: a single-cell data platform for scalable exploration, analysis and modeling of aggregated data CZI Single-Cell Biology Program. 10.1101/2023.10.30.563174 PMC11701654 39607691

[B2] Adil A. Kumar V. Jan A. T. Asger M. (2021). Single-cell transcriptomics: current methods and challenges in data acquisition and analysis. In Front. Neurosci., (Vol. 15). 591122, Frontiers Media S.A. 10.3389/fnins.2021.591122 33967674 PMC8100238

[B3] Aguet F. Anand S. Ardlie K. G. Gabriel S. Getz G. A. Graubert A. (2020). The GTEx Consortium atlas of genetic regulatory effects across human tissues. Science 369 (6509), 1318–1330. 10.1126/science.aaz1776 32913098 PMC7737656

[B4] Ahmed J. Alaba O. Ameen G. Arora V. Arteaga-Vazquez M. A. Arun A. (2021). Vision, challenges and opportunities for a plant cell atlas. ELife 10, e66877. 10.7554/eLife.66877 34491200 PMC8423441

[B5] Narula S. Jain A. Ameen G. Prachi (2015). Cloud computing security: Amazon web service. 2015 Fifth International Conference on Advanced Computing and Communication Technologies, 501–505. 10.1109/ACCT.2015.20

[B6] Athar A. Füllgrabe A. George N. Iqbal H. Huerta L. Ali A. (2019). ArrayExpress update – from bulk to single-cell expression data. Nucleic Acids Res. 47 (D1), D711-D715–D715. 10.1093/nar/gky964 30357387 PMC6323929

[B7] Azevedo R. de M. Dumontier M. (2020). Considerations for the conduction and interpretation of fairness evaluations. Data Intell. 2 (1–2), 285–292. 10.1162/dint_a_00051

[B8] Bisong E. (2019). “An overview of Google cloud platform services,” in Building machine learning and deep learning models on Google cloud platform, 7–10. Apress. 10.1007/978-1-4842-4470-8_2

[B9] Buels R. Yao E. Diesh C. M. Hayes R. D. Munoz-Torres M. Helt G. (2016). JBrowse: a dynamic web platform for genome visualization and analysis. Genome Biol. 17 (1), 66. 10.1186/s13059-016-0924-1 27072794 PMC4830012

[B10] Chen L. Li H. Teng J. Wang Z. Qu X. Chen Z. (2023). Construction of a multi-tissue cell atlas reveals cell-type-specific regulation of molecular and complex phenotypes in pigs. BioRxiv. 10.1101/2023.06.12.544530 PMC12884822 41309493

[B11] Childs A. Chand D. Pereira J. Santra S. Rajaraman S. (2024). BacteSign: building a findable, accessible, interoperable, and reusable (FAIR) database for universal bacterial identification. Biosensors 14 (4), 176. 10.3390/bios14040176 38667169 PMC11047924

[B12] Clark E. L. Archibald A. L. Daetwyler H. D. Groenen M. A. M. Harrison P. W. Houston R. D. (2020). From FAANG to fork: application of highly annotated genomes to improve farmed animal production. Genome Biol. 21 (1), 285. 10.1186/s13059-020-02197-8 33234160 PMC7686664

[B13] Clough E. Barrett T. (2016). The gene expression omnibus database. Methods Mol. Biol. 1418, 93–110. 10.1007/978-1-4939-3578-9_5 27008011 PMC4944384

[B14] Cole B. Bergmann D. Blaby-Haas C. E. Blaby I. K. Bouchard K. E. Brady S. M. (2021). Plant single-cell solutions for energy and the environment. Commun. Biol. 4 (1), 962. 10.1038/s42003-021-02477-4 34385583 PMC8361165

[B15] Dunn N. A. Unni D. R. Diesh C. Munoz-Torres M. Harris N. L. Yao E. (2019). Apollo: democratizing genome annotation. PLOS Comput. Biol. 15 (2), e1006790. 10.1371/journal.pcbi.1006790 30726205 PMC6380598

[B16] Duyzend M. H. Cacheiro P. Jacobsen J. O. B. Giordano J. Brand H. Wapner R. J. (2024). Improving prenatal diagnosis through standards and aggregation. Prenat. Diagn. 44 (4), 454–464. 10.1002/pd.6522 38242839 PMC11006584

[B17] Fahlgren N. Kapoor M. Yordanova G. Papatheodorou I. Waese J. Cole B. (2023). Toward a data infrastructure for the plant cell atlas. Plant Physiol. 191 (1), 35–46. 10.1093/plphys/kiac468 36200899 PMC9806565

[B18] Geewax J. J. (2018). Google Cloud platform in action. Manning Publications.

[B19] Giuffra E. Tuggle C. K. (2019). Functional annotation of animal genomes (FAANG): current achievements and roadmap. Annu. Rev. Animal Biosci. 7 (1), 65–88. 10.1146/annurev-animal-020518-114913 30427726

[B20] Grones C. Eekhout T. Shi D. Neumann M. Berg L. S. Ke Y. (2024). Best practices for the execution, analysis, and data storage of plant single-cell/nucleus transcriptomics. Plant Cell 36 (Issue 4), 812–828. 10.1093/plcell/koae003 38231860 PMC10980355

[B21] Hall J. L. Honeycutt S. Gonzalez N. O’Donnell-Luria A. Overby Taylor C. Stevens L. (2023). National human genome research Institute genomic data science analysis, visualization, and informatics lab-space: reaching out to clinicians. Circulation Genomic Precis. Med. 16 (3), 275–276. 10.1161/CIRCGEN.122.003936 PMC10619961 37013830

[B22] Haniffa M. Taylor D. Linnarsson S. Aronow B. J. Bader G. D. Barker R. A. (2021). A roadmap for the human developmental cell atlas. Nature 597 (7875), 196–205. 10.1038/s41586-021-03620-1 34497388 PMC10337595

[B23] Harrison P. W. Fan J. Richardson D. Clarke L. Zerbino D. Cochrane G. (2018). FAANG, establishing metadata standards, validation and best practices for the farmed and companion animal community. Anim. Genet. 49 (6), 520–526. 10.1111/age.12736 30311252 PMC6334167

[B24] Harrison P. W. Sokolov A. Nayak A. Fan J. Zerbino D. Cochrane G. (2021). The FAANG data portal: global, open-access, “FAIR”, and richly validated genotype to phenotype data for high-quality functional annotation of animal genomes. Front. Genet. 12, 639238. 10.3389/fgene.2021.639238 34220930 PMC8248360

[B25] He Z. Luo Y. Zhou X. Zhu T. Lan Y. Chen D. (2024). scPlantDB: a comprehensive database for exploring cell types and markers of plant cell atlases. Nucleic Acids Res. 52 (D1), D1629–D1638. 10.1093/nar/gkad706 37638765 PMC10767885

[B26] Herrera-Uribe J. Lim K. S. Byrne K. A. Daharsh L. Liu H. Corbett R. J. (2023). Integrative profiling of gene expression and chromatin accessibility elucidates specific transcriptional networks in porcine neutrophils. Front. Genet. 14, 1107462. 10.3389/fgene.2023.1107462 37287538 PMC10242145

[B27] Herrera-Uribe J. Wiarda J. E. Sivasankaran S. K. Daharsh L. Liu H. Byrne K. A. (2021). Reference transcriptomes of porcine peripheral immune cells created through bulk and single-cell RNA sequencing. Front. Genet. 12, 689406. 10.3389/fgene.2021.689406 34249103 PMC8261551

[B68] Hovig E. Gundersen S. Boddu S. Capella-Gutierrez S. Drabløs F. Fernández J. M. (2021). Recommendations for the FAIRification of genomic track metadata. F1000Research. 10. 10.12688/f1000research.28449.1 PMC8226415 34249331

[B28] Hu H. Miao Y.-R. Jia L.-H. Yu Q.-Y. Zhang Q. Guo A.-Y. (2019). AnimalTFDB 3.0: a comprehensive resource for annotation and prediction of animal transcription factors. Nucleic Acids Res. 47 (D1), D33-D38–D38. 10.1093/nar/gky822 30204897 PMC6323978

[B29] Huynh-Thu V. A. Irrthum A. Wehenkel L. Geurts P. (2010). Inferring regulatory networks from expression data using tree-based methods. PLoS ONE 5 (9), e12776. 10.1371/journal.pone.0012776 20927193 PMC2946910

[B30] Jacobsen A. Kaliyaperumal R. Santos L. O. B. da S. Mons B. Schultes E. Roos M. (2020). A generic workflow for the data fairification process. Data Intell. 2 (1–2), 56–65. 10.1162/dint_a_00028

[B31] Jha S. G. Borowsky A. T. Cole B. J. Fahlgren N. Farmer A. Huang S. S. C. (2021). Vision, challenges and opportunities for a plant cell atlas. ELife 10, e66877. 10.7554/eLife.66877 34491200 PMC8423441

[B32] Leinonen R. Akhtar R. Birney E. Bower L. Cerdeno-Tarraga A. Cheng Y. (2011a). The European nucleotide archive. Nucleic Acids Res. 39, D28–D31. 10.1093/nar/gkq967 20972220 PMC3013801

[B33] Leinonen R. Sugawara H. Shumway M. (2011b). The sequence read archive. Nucleic Acids Res. 39, D19–D21. 10.1093/nar/gkq1019 21062823 PMC3013647

[B34] Li H. Janssens J. De Waegeneer M. Kolluru S. S. Davie K. Gardeux V. (2022). Fly Cell Atlas: a single-nucleus transcriptomic atlas of the adult fruit fly. Science 375 (6584), eabk2432. 10.1126/science.abk2432 35239393 PMC8944923

[B35] Lun A. T. L. Riesenfeld S. Andrews T. Dao T. P. Gomes T. Marioni J. C. (2019). EmptyDrops: distinguishing cells from empty droplets in droplet-based single-cell RNA sequencing data. Genome Biol. 20 (1), 63. 10.1186/s13059-019-1662-y 30902100 PMC6431044

[B36] Malone J. Holloway E. Adamusiak T. Kapushesky M. Zheng J. Kolesnikov N. (2010). Modeling sample variables with an experimental factor ontology. Bioinformatics 26 (8), 1112–1118. 10.1093/bioinformatics/btq099 20200009 PMC2853691

[B37] Moreno P. Fexova S. George N. Manning J. R. Miao Z. Mohammed S. (2022). Expression Atlas update: gene and protein expression in multiple species. Nucleic Acids Res. 50 (D1), D129–D140. 10.1093/nar/gkab1030 34850121 PMC8728300

[B38] Neubert E. N. DeRogatis J. M. Lewis S. A. Viramontes K. M. Ortega P. Henriquez M. L. (2023). HMGB2 regulates the differentiation and stemness of exhausted CD8+ T cells during chronic viral infection and cancer. Nat. Commun. 14 (1), 5631. 10.1038/s41467-023-41352-0 37704621 PMC10499904

[B39] O’Connor B. D. Yuen D. Chung V. Duncan A. G. Liu X. K. Patricia J. (2017). The Dockstore: enabling modular, community-focused sharing of Docker-based genomics tools and workflows. F1000Research 6, 52. 10.12688/f1000research.10137.1 28344774 PMC5333608

[B40] Osumi-Sutherland D. Xu C. Keays M. Levine A. P. Kharchenko P. V. Regev A. (2021). Cell type ontologies of the human cell atlas. Nat. Cell Biol. 23 (11), 1129–1135. 10.1038/s41556-021-00787-7 34750578

[B41] Palankar M. R. Iamnitchi A. Ripeanu M. Garfinkel S. (2008). “Amazon S3 for science grids,” in Proceedings of the 2008 international workshop on data-aware distributed computing, 55–64. 10.1145/1383519.1383526

[B42] Papatheodorou I. Moreno P. Manning J. Fuentes A. M.-P. George N. Fexova S. (2019). Expression Atlas update: from tissues to single cells. Nucleic Acids Res. 48, D77-D83. 10.1093/nar/gkz947 PMC7145605 31665515

[B43] Parkinson H. Kapushesky M. Shojatalab M. Abeygunawardena N. Coulson R. Farne A. (2007). ArrayExpress--a public database of microarray experiments and gene expression profiles. Nucleic Acids Res. 35, D747–D750. 10.1093/nar/gkl995 17132828 PMC1716725

[B44] Perkel J. M. (2022). Terra takes the pain out of ‘omics’ computing in the cloud. Nature 601 (7891), 154–155. 10.1038/d41586-021-03822-7 34983991

[B45] Perrone G. Unpingco J. Lu H.-M. (2020). Network visualizations with Pyvis and VisJS. ArXiv Preprint ArXiv:2006.04951.

[B46] Raney B. J. Barber G. P. Benet-Pagès A. Casper J. Clawson H. Cline M. S. (2024). The UCSC Genome Browser database: 2024 update. Nucleic Acids Res. 52 (D1), D1082–D1088. 10.1093/nar/gkad987 37953330 PMC10767968

[B47] Rayner T. F. Rocca-Serra P. Spellman P. T. Causton H. C. Farne A. Holloway E. (2006). A simple spreadsheet-based, MIAME-supportive format for microarray data: MAGE-TAB. BMC Bioinforma. 7 (1), 489. 10.1186/1471-2105-7-489 PMC1687205 17087822

[B48] Regev A. Teichmann S. Rozenblatt-Rosen O. Stubbington M. Ardlie K. Amit I. (2018). The human cell atlas white paper.

[B49] Regev A. Teichmann S. A. Lander E. S. Amit I. Benoist C. Birney E. (2017). The human cell atlas. ELife 6, e27041. 10.7554/eLife.27041 29206104 PMC5762154

[B50] Rhee S. Y. Birnbaum K. D. Ehrhardt D. W. (2019). Towards building a plant cell atlas. Trends Plant Sci. 24 (4), 303–310. 10.1016/j.tplants.2019.01.006 30777643 PMC7449582

[B51] Rozenblatt-Rosen O. Shin J. W. Rood J. E. Hupalowska A. Regev A. Heyn H. (2021). Building a high-quality human cell atlas. Nat. Biotechnol. 39 (2), 149–153. 10.1038/s41587-020-00812-4 33500565

[B52] Sarkans U. Gostev M. Athar A. Behrangi E. Melnichuk O. Ali A. (2018). The BioStudies database—one stop shop for all data supporting a life sciences study. Nucleic Acids Res. 46 (D1), D1266-D1270–D1270. 10.1093/nar/gkx965 29069414 PMC5753238

[B53] Sheffield N. C. Bonazzi V. R. Bourne P. E. Burdett T. Clark T. Grossman R. L. (2022). From biomedical cloud platforms to microservices: next steps in FAIR data and analysis. Sci. Data 9 (1), 553. 10.1038/s41597-022-01619-5 36075919 PMC9458632

[B54] Sheffield N. C. LeRoy N. J. Khoroshevskyi O. (2023). Challenges to sharing sample metadata in computational genomics. Front. Genet. 14, 1154198. 10.3389/fgene.2023.1154198 37287537 PMC10243526

[B55] Speir M. L. Bhaduri A. Markov N. S. Moreno P. Nowakowski T. J. Papatheodorou I. (2021). UCSC Cell Browser: visualize your single-cell data. Bioinformatics 37 (23), 4578–4580. 10.1093/bioinformatics/btab503 34244710 PMC8652023

[B56] Tekman M. Batut B. Ostrovsky A. Antoniewski C. Clements D. Ramirez F. (2020). A single-cell RNA-sequencing training and analysis suite using the Galaxy framework. GigaScience 9 (10), giaa102. 10.1093/gigascience/giaa102 33079170 PMC7574357

[B57] Tello-Ruiz M. K. Naithani S. Gupta P. Olson A. Wei S. Preece J. (2021). Gramene 2021: harnessing the power of comparative genomics and pathways for plant research. Nucleic Acids Res. 49 (D1), D1452–D1463. 10.1093/nar/gkaa979 33170273 PMC7779000

[B58] Thelwall M. Kousha K. (2016). Figshare: a universal repository for academic resource sharing? Online Inf. Rev. 40 (3), 333–346. 10.1108/OIR-06-2015-0190

[B59] Thompson M. Burger K. Kaliyaperumal R. Roos M. da Silva Santos L. O. B. (2020). Making FAIR easy with FAIR tools: from creolization to convergence. Data Intell. 2 (1–2), 87–95. 10.1162/dint_a_00031

[B60] Tixier-Boichard M. Fabre S. Dhorne-Pollet S. Goubil A. Acloque H. Vincent-Naulleau S. (2021). Tissue resources for the functional annotation of animal genomes. Front. Genet. 12, 666265. 10.3389/fgene.2021.666265 34234809 PMC8256271

[B61] Tuggle C. K. Giuffra E. White S. N. Clarke L. Zhou H. Ross P. J. (2016). GO ‐ FAANG meeting: a gathering on functional annotation of an imal genomes. Anim. Genet. 47 (5), 528–533. 10.1111/age.12466 27453069 PMC5082551

[B62] Wang Z. Lachmann A. Ma’ayan A. (2019). Mining data and metadata from the gene expression omnibus. Biophys. Rev. 11 (1), 103–110. 10.1007/s12551-018-0490-8 30594974 PMC6381352

[B63] Weigel T. Schwardmann U. Klump J. Bendoukha S. Quick R. (2020). Making data and workflows findable for machines. Data Intell. 2 (1–2), 40–46. 10.1162/dint_a_00026

[B64] Wilder B. (2012). Cloud architecture patterns: using Microsoft azure. O’Reilly Media, Inc.

[B65] Wilkinson M. D. Dumontier M. Aalbersberg Ij. J. Appleton G. Axton M. Baak A. (2016). The FAIR Guiding Principles for scientific data management and stewardship. Sci. Data 3 (1), 160018. 10.1038/sdata.2016.18 26978244 PMC4792175

[B66] Wilkinson M. D. Dumontier M. Sansone S. A. Bonino da Silva Santos L. O. Prieto M. Batista D. (2019). Evaluating FAIR maturity through a scalable, automated, community-governed framework. Sci. Data 6 (1), 174. 10.1038/s41597-019-0184-5 31541130 PMC6754447

[B67] Wilkinson M. D. Sansone S.-A. Schultes E. Doorn P. Olavo L. Da B. (2018). Comment: a design framework and exemplar metrics for FAIRness. Nature Publishing Group. ( https://pubmed.ncbi.nlm.nih.gov/26978244/ ). 10.25504/FAIRsharing.WWI10U PMC6018520 29944145

