# Supplementary material for: Building a FAIR data ecosystem for incorporating single-cell transcriptomics data into agricultural genome to phenome research
Source: Front Genet. 2024 Nov 29;15:1460351. doi: 10.3389/fgene.2024.1460351 (PMC11638175; doi:10.3389/fgene.2024.1460351)
Supplement: Supplementary file 1 [file Table1.docx]

Supplementary Material

# Supplementary Data

Supplementary tables consist of updated single cell rulesets within FAANG data portal and list of plant ingested datasets in the single cell expression atlas.

## Supplementary Tables

**Supplementary Table 1.** Updated single-cell rulesets in FAANG- Data portal to perform ingestion in HCA-Data portal service.

| **Single Cell RNA Sequencing** | **Single Cell ATAC Sequencing** |
| --- | --- |
| experiment target | experiment target |
| library construction | transposase protocol |
| primer | transposed DNA sequence file read index |
| amplification method | cell barcode read |
| amplification cycles | sample index read |
| end bias | nucleic acid molecule |
| library strand | nucleic acid source |
| spike in | sequencing method |
| spike in dilution or concentration | kit retail name |
| library generation protocol | kit manufacturer |
| sequencing protocol | sequencing protocol |
| read strand | library construction method |
| rna purity 260:280 ratio |  |
| rna purity 260:230 ratio |  |
| rna integrity number |  |

**Supplementary Table 2.** Plant single-cell expression data derived from 20 experimental studies from 4 major plant genomes (Arabidopsis thaliana, Oryza sativa, Solanum lycopersicum, Zea mays) (Supplementary table 2) for ingestion to EMBL-EBI single-cell expression atlas.

| **Species** | **Title** | **Sample Metadata** | **Conditions/ Treatments** | **Number of cells** | **Pubmed ID** | **DOI id** | **Publication links** |
| --- | --- | --- | --- | --- | --- | --- | --- |
| Arabidopsis thaliana | Single-cell RNA-seq analysis reveals ploidy-dependent and cell-specific transcriptome changes in Arabidopsis female gametophytes. | Female gametic cell | Development | 46 | 32698836 | <https://doi.org/10.1186/s13059-020-02094-0> | *Song Q, Ando A, Jiang N, Ikeda Y, Chen ZJ. et al. (2020) Single-cell RNA-seq analysis reveals ploidy-dependent and cell-specific transcriptome changes in Arabidopsis female gametophytes.* |
| Arabidopsis thaliana | Single-cell transcriptomics sheds light on the identity and metabolism of developing Arabidopsis leaf cells. | Leaf | Light stress | 634 | 34687312 | <https://doi.org/10.1093/plphys/kiab489> | *Tenorio Berrío R, Verstaen K, Vandamme N, Pevernagie J, Achon I et al. (2022) Single-cell transcriptomics sheds light on the identity and metabolism of developing leaf cells.* |
| Arabidopsis thaliana | Distinct identities of leaf phloem cells revealed by single cell transcriptomics. | Leaf | Development | 4837 | 33955487 | [10.1093/plcell/koaa060](https://doi.org/10.1093/plcell/koaa060) | *Kim JY, Symeonidi E, Pang TY, Denyer T, Weidauer D et al. (2021) Distinct identities of leaf phloem cells revealed by single cell transcriptomics.* |
| Arabidopsis thaliana | A single cell Arabidopsisroot atlas reveals developmental trajectories in wild type and cell identity mutants | Roots | Development | 361,743 | 35134336 | [10.1016/j.devcel.2022.01.008](https://doi.org/10.1016/j.devcel.2022.01.008) | *Shahan R, Hsu C, Nolan TM, Cole BJ, Taylor IW et al. (2020) A single cellArabidopsisroot atlas reveals developmental trajectories in wild type and cell identity mutants* |
| Arabidopsis thaliana | Vascular transcription factors guide plant epidermal responses to limiting phosphate | Roots | Limiting Phosphorous | 14961 | 32943451 | <https://doi.org/10.1126/science.aay4970> | *Wendrich JR, Yang B, Vandamme N, Verstaen K, Smet W et al. (2020) Vascular transcription factors guide plant epidermal responses to limiting phosphate conditions.* |
| Arabidopsis thaliana | A single cell view of the transcriptome during lateral root initiation inArabidopsis thaliana | Roots | Development | 9090 | 33822225 | <https://doi.org/10.1093/plcell/koab101> | *Gala HP, Lanctot A, Jean-Baptiste K, Guiziou S, Chu JC et al. (2020) A single cell view of the transcriptome during lateral root initiation inArabidopsis thaliana* |
| Arabidopsis thaliana | Single-Cell RNA Sequencing Resolves Molecular Relationships Among Individual Plant Cells. | Roots | Development | 46991 | 30718350 | <https://doi.org/10.1104/pp.18.01482> | *Ryu KH, Huang L, Kang HM, Schiefelbein J. (2019) Single-Cell RNA Sequencing Resolves Molecular Relationships Among Individual Plant Cells.* |
| Arabidopsis thaliana | Dynamics of Gene Expression in Single Root Cells of Arabidopsis thaliana. | Roots | Development | 3121 | 30923229 | <https://doi.org/10.1105/tpc.18.00785> | *Jean-Baptiste K, McFaline-Figueroa JL, Alexandre CM, Dorrity MW, Saunders L et al. (2019) Dynamics of Gene Expression in Single Root Cells of Arabidopsis thaliana.* |
| Arabidopsis thaliana | High-Throughput Single-Cell Transcriptome Profiling of Plant Cell Types. | Roots | Development | 3552 | 31091459 | <https://doi.org/10.1016/j.celrep.2019.04.054> | *Shulse CN, Cole BJ, Ciobanu D, Lin J, Yoshinaga Y et al. (2019) High-Throughput Single-Cell Transcriptome Profiling of Plant Cell Types.* |
| Arabidopsis thaliana | High-Throughput Single-Cell Transcriptome Profiling of Plant Cell Types. | Roots | Development | 10779 | 31091459 | <https://doi.org/10.1016/j.celrep.2019.04.054> | *Shulse CN, Cole BJ, Ciobanu D, Lin J, Yoshinaga Y et al. (2019) High-Throughput Single-Cell Transcriptome Profiling of Plant Cell Types.* |
| Arabidopsis thaliana | Molecular Mechanisms Driving Switch Behavior in Xylem Cell Differentiation. | Roots | Development | 6727 | 31291572 | <https://doi.org/10.1016/j.celrep.2019.06.041> | *Turco GM, Rodriguez-Medina J, Siebert S, Han D, Valderrama-Gómez MÁ et al. (2019) Molecular Mechanisms Driving Switch Behavior in Xylem Cell Differentiation.* |
| Arabidopsis thaliana | Global Dynamic Molecular Profiling of Stomatal Lineage Cell Development by Single-Cell RNA Sequencing. | Seedling Cotyledons | Development | 46997 | 32592820 | <https://doi.org/10.1016/j.molp.2020.06.010> | *Liu Z, Zhou Y, Guo J, Li J, Tian Z et al. (2020) Global Dynamic Molecular Profiling of Stomatal Lineage Cell Development by Single-Cell RNA Sequencing.* |
| Arabidopsis thaliana | A single-cell analysis of the Arabidopsis vegetative shoot apex. | Shoot apex | Development | 53581 | 33725481 | <https://doi.org/10.1016/j.devcel.2021.02.021> | *Zhang TQ, Chen Y, Wang JW. (2021) A single-cell analysis of the Arabidopsis vegetative shoot apex.* |
| Oryza sativa Indica Group | Single-cell transcriptomic analysis of rice root tips – Oryza sativa Indica Group | Roots | Development | 9093 | 33352304 | <https://doi.org/10.1016/j.molp.2020.12.014> | *Liu Q, Liang Z, Feng D, Jiang S, Wang Y et al. (2021) Transcriptional landscape of rice roots at the single-cell resolution.* |
| Oryza sativa Japonica Group | Single-cell transcriptome atlas and chromatin accessibility landscape reveal differentiation trajectories in the rice root. | Roots | Development | 28857 | 33824350 | <https://doi.org/10.1038/s41467-021-22352-4> | *Zhang TQ, Chen Y, Liu Y, Lin WH, Wang JW. et al. (2021) Single-cell transcriptome atlas and chromatin accessibility landscape reveal differentiation trajectories in the rice root.* |
| Oryza sativa Japonica Group | Single-cell transcriptomic analysis of rice root tips – Oryza sativa Japonica Group | Roots | Development | 10999 | 33352304 | <https://doi.org/10.1016/j.molp.2020.12.014> | *Liu Q, Liang Z, Feng D, Jiang S, Wang Y et al. (2021) Transcriptional landscape of rice roots at the single-cell resolution.* |
| Solanum lycopersicum | Single-nucleus RNA-seq resolves spatiotemporal developmental trajectories in the tomato shoot apex | Shoot apex | Development | 5254 |  | https://www.biorxiv.org/content/10.1101/2020.09.20.305029v1 | *Tian C, Du Q, Xu M, Du F, Jiao Y. et al. (2020) Single-nucleus RNA-seq resolves spatiotemporal developmental trajectories in the tomato shoot apex* |
| Solanum lycopersicum | Single-nucleus RNA-seq resolves spatiotemporal developmental trajectories in the tomato shoot apex | Shoot apex | Development | 22572 |  | https://www.biorxiv.org/content/10.1101/2020.09.20.305029v1 | *Tian C, Du Q, Xu M, Du F, Jiao Y. et al. (2020) Single-nucleus RNA-seq resolves spatiotemporal developmental trajectories in the tomato shoot apex* |
| Zea mays | Single-cell RNA sequencing of developing maize ears facilitates functional analysis and trait candidate gene | Inflorescence | Development | 12525 | 33400914 | <https://doi.org/10.1016/j.devcel.2020.12.015> | *Xu X, Crow M, Rice BR, Li F, Harris B et al. (2021) Single-cell RNA sequencing of developing maize ears facilitates functional analysis and trait candidate gene discovery.* |
| Zea mays | Evidence for phloem loading via the abaxial bundle sheath cells in maize leaves. | Leaf | Development | 10901 | 33955497 | <https://doi.org/10.1093/plcell/koaa055> | *Bezrutczyk M, Zöllner NR, Kruse CPS, Hartwig T, Lautwein T et al. (2021) Evidence for phloem loading via the abaxial bundle sheath cells in maize leaves.* |
